# Impact of Structured Surveillance and Intervention on Long-Term Vein Outcomes in Pediatric Pulmonary Vein Stenosis

**DOI:** 10.3390/children13070933

**Published:** 2026-07-16

**Authors:** Ashish Saini, Andrew Jergel, Yijin Xiang, Rosemary Gray, Dennis W. Kim, James A. Kuo

**Affiliations:** 1 Division of Pediatric Cardiology, Children’s Healthcare of Atlanta, Emory University School of Medicine, Atlanta, GA 30329, USAkimd@kidsheart.com (D.W.K.);; 2 Pediatric Biostatistics Core, Emory University School of Medicine, Atlanta, GA 30307, USAyijinxia@usc.edu (Y.X.)

**Keywords:** pediatric pulmonary vein stenosis, primary pulmonary vein stenosis, congenital heart disease, interventional cardiology

## Abstract

**Highlights:**

**What are the main findings?**

- Most pulmonary vein loss (60%) occurs within 3 years of diagnosis, peaking in year 1.
- Veins with recurrent disease that remain patent beyond 3 years require fewer reinterventions for sustained patency.

**What is the implication of the main findings?**

- The first 3 years after diagnosis represent the highest risk period for vein loss.
- Early aggressive surveillance and interventions during this period may improve long-term outcomes.

**Abstract:**

**Background:** Pediatric pulmonary vein stenosis (PVS) is a progressive and recurrent condition associated with high morbidity and mortality. Long-term outcomes of individual pulmonary veins remain poorly described. **Methods:** All pediatric (<18 years) PVS cases diagnosed between September 2005 and January 2020 with a minimum follow-up of 5 years were included. Vein-specific outcomes were assessed using competing-risk survival analysis adjusted for patient death and vein atresia. Veins with recurrent disease maintaining patency for ≥3 years were analyzed longitudinally to characterize reintervention burden as a surrogate for disease trajectory. **Results:** A total of 244 affected veins in 107 patients were identified. The cumulative incidence of vein loss progressively increased during the first 3 years, followed by plateauing thereafter. Overall, 147 of 244 veins (60%) were lost within 3 years of diagnosis, including 75 veins lost secondary to patient death. Vein attrition was greatest during the first year following diagnosis. Ninety-seven veins remained patent for ≥3 years, including 73 with recurrent disease. Among these veins, the median reintervention rate decreased significantly from 2 (IQR: 1–4) during the first 3 years to 1 per year (IQR: 1–3) thereafter (*p* < 0.01) while maintaining continued patency. **Conclusions:** In pediatric PVS, the first 3 years after diagnosis represent a critical period of active disease with high rates of vein attrition. Veins maintaining patency beyond this period demonstrate a reduced reintervention rate, suggesting stabilization of disease progression. Aggressive surveillance and intervention during this therapeutic window may improve long-term vein and patient outcomes.

## 1. Introduction

Pediatric pulmonary vein stenosis (PVS) is a heterogeneous condition characterized by a progressive and recurrent nature, leading to adverse clinical outcomes. Pediatric PVS is classified as primary when occurring in a native pulmonary vein or secondary when following a surgical intervention for total or partial anomalous pulmonary venous connection (TAPVC or PAPVC). The incidence of primary PVS is approximately 1.7 per 100,000 children, with prematurity, low birth weight, left to right shunts and genetic syndromes being significant risk factors [1]. This is likely an underestimate in the current era of enhanced surveillance and improved survival of this vulnerable population.

The recurrent nature of PVS has been attributed to intimal hyperplasia with myofibroblast proliferation [2]. Additional mechanisms include anatomic distortion from the surrounding anatomy such as the left upper pulmonary vein by left posterior bronchus, left lower pulmonary vein between the cardiac mass and aorta, and right upper pulmonary vein by the right pulmonary artery [3]. Secondary PVS can occur due to residual atrial tissue, surgical scar, or inadequate surgical confluence anastomosis [4]. Management strategy for pediatric PVS has evolved to include a combination of surgical, transcatheter and medical therapy. Using this multimodal approach, our institution has developed a dedicated pulmonary vein rehabilitation program.

Prior studies have largely focused on patient-level outcomes, whereas the long-term trajectory of individual pulmonary veins remains poorly understood. Improved understanding of long-term vein-specific outcomes may help guide surveillance intervals and optimize timing of interventions. This study sought to evaluate the long-term outcomes of individual pulmonary veins in pediatric patients with PVS managed within a structured pulmonary vein rehabilitation program. Specifically, we aimed to characterize vein attrition and the temporal pattern of disease activity in veins with recurrent disease using longitudinal reintervention rate as a surrogate for disease activity.

## 2. Materials and Methods

### 2.1. Subjects

The study is a retrospective review of patients younger than 18 years of age diagnosed with and treated for pulmonary vein stenosis at Children’s Healthcare of Atlanta between September 2005 and January 2020 with a minimum follow-up duration of 5 years. Patients were identified from the institutional pulmonary vein rehabilitation program database and included patients with both primary and secondary PVS. The Children’s Healthcare of Atlanta Institutional Review Board approved the study and waived the need for parental consent (IRB # 00000919).

### 2.2. Data Collection

Patient demographics, clinical details, vein characteristics, intervention data, and outcomes were collected through medical records review. Affected veins were defined as pulmonary veins with luminal narrowing demonstrated by echocardiographic, computed tomography, or angiographic imaging. The outcome of individual veins was characterized as either patent or vein loss. Vein loss occurred due to atresia or patient death. Using survival analysis, adjusted for competing risks of patient death and vein atresia, stabilization in the incidence of vein attrition was identified three years after diagnosis. Accordingly, to evaluate the long-term disease trajectory of individual veins, we isolated a conditional cohort of veins with recurrent disease that maintained luminal patency for ≥3 years from baseline diagnosis. Recurrent disease was defined as the need for two or more interventions during the first 12 months of diagnosis to sustain luminal patency. Within this cohort, the reintervention rate for individual veins, defined as the number of transcatheter and surgical interventions performed per year, was serially analyzed and used as a surrogate for disease trajectory.

**Figure 1:**
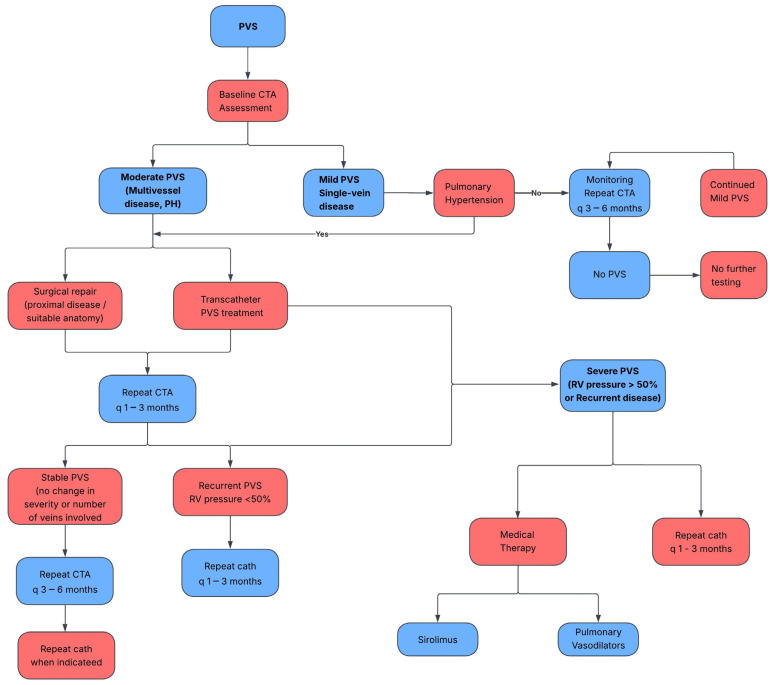
Management Algorithm for Pulmonary Vein Rehabilitation Program. CTA: computed tomography angiography; PH: pulmonary hypertension; PVS: pulmonary vein stenosis; RV: right ventricle.

At our institution, all patients with PVS are managed within a dedicated pulmonary vein rehabilitation program utilizing standardized surveillance and intervention protocols that incorporate transcatheter, surgical and adjunctive medical therapy (Figure 1). Interventions are guided by objective clinical and hemodynamic criteria, including worsening clinical status, disease progression on imaging, and increasing right ventricular systolic pressure, which trigger reintervention. All patients with suspected pulmonary vein stenosis undergo baseline computed tomography (CT) angiography to define disease extent and anatomy. Patients with mild, single-vessel disease undergo serial surveillance, whereas those with multivessel, progressive, or symptomatic disease undergo intervention. Transcatheter therapy is the primary treatment modality for most patients. Surgical repair is reserved for patients with recurrent pulmonary vein stenosis following anomalous pulmonary venous connection repair and favorable anatomy, or those with previously implanted stents that cannot be further dilated or intentionally fractured. In selected cases, pulmonary vein stents are implanted intraoperatively to facilitate future transcatheter redilation.

Catheter-based therapy is individualized according to vessel size, lesion characteristics, disease extent, and clinical status. Balloon angioplasty with conventional and drug-coated balloons is the preferred initial treatment for small pulmonary veins, while cutting balloons are reserved for resistant lesions. Based on our institutional experience, practice has evolved from routine stenting toward aggressive serial balloon angioplasty with selective stent implantation for larger vessels or disease refractory to balloon angioplasty. When stents are used, care is taken to preserve side branches and maintain future redilation potential. The stents currently employed at our institution include drug-eluting coronary stents for vessel diameter < 5mm and pre-mounted peripheral stents for 5–7 mm vessel diameter.

Systemic sirolimus is the preferred antiproliferative therapy at our institution and is considered for patients with recurrent multivessel PVS and right ventricular (RV) hypertension. The timing of initiation is individualized according to disease severity and clinical course but may begin immediately after cardiac catheterization or at least two weeks following surgical intervention. Therapy is administered in collaboration with pediatric hematology. Pulmonary vasodilators are selectively used based on clinical trajectory, respiratory status, comorbidities, and hemodynamic evaluation demonstrating elevated pulmonary vascular resistance after rehabilitation of affected veins or responsiveness to acute vasodilator testing. Pulmonary vasodilator therapy is directed by our institution’s pulmonary hypertension program. Importantly, decisions are directed by program leadership in accordance with established institutional protocols and multidisciplinary discussion when indicated, thereby minimizing inter-operator variability.

### 2.3. Statistical Analysis

Continuous variables were reported as median with interquartile ranges (IQR), whereas categorical variables were reported as frequencies and percentages. Time-to-vein loss was analyzed using Kaplan–Meier analysis with cumulative incidence models to adjust for the competing risks of patient death and vein atresia. Univariable sub-distribution hazard modeling using Fine–Gray regression, clustered on multiple veins within individual patients, was utilized to compare potential confounding variables between veins that were lost and the cohort of long-term patent veins with recurrent disease. Finally, reintervention rate was visualized using box–whisker plots, with pairwise statistical comparisons performed using the Mann–Whitney U test. For all statistical tests and comparisons, a *p*-value of <0.05 was considered statistically significant. Data cleaning, analysis, and visualization were performed using R Statistical Software (version 4.2.1; R Core Team, 2022).

## 3. Results

A total of 244 affected veins in 107 patients were identified during the study period. Seventy-seven patients (72%) were diagnosed before 6 months of age, with a median age at diagnosis of 4.1 months (IQR: 2.2–6.4 months); 54% were female. The median gestational age at birth was 35 weeks (IQR: 28–38), with 30 (28%) born before 32 weeks. Bronchopulmonary dysplasia occurred in 48 (45%) patients. Nineteen patients (18%) had associated genetic syndromes. Eighty patients (75%) had primary PVS, 47 of whom had associated congenital heart disease (CHD) and 33 had normal cardiac anatomy. Left to right shunt lesions such as atrial septal defect (ASD), ventricular septal defect (VSD) and complete atrioventricular canal defect were the predominant CHD diagnoses. Twenty-seven patients (25%) had partial or total anomalous pulmonary venous connection. At diagnosis, 90 patients (84%) had multivessel disease. The overall mortality rate was 30%. The median age at death was 13.5 months (IQR: 7.6–29.6), with a median interval of 9.5 months (IQR: 5.6–21.7) from PVS diagnosis to death. Among the 244 affected veins, 168 (69%) had primary PVS, while the remainder were secondary. Left-sided veins were more frequently affected, accounting for 58% of the affected veins, with the left upper pulmonary vein being the most affected (76/244, 31%). Table 1 and Table 2 summarize baseline patient and vein characteristics.

**Figure 2:**
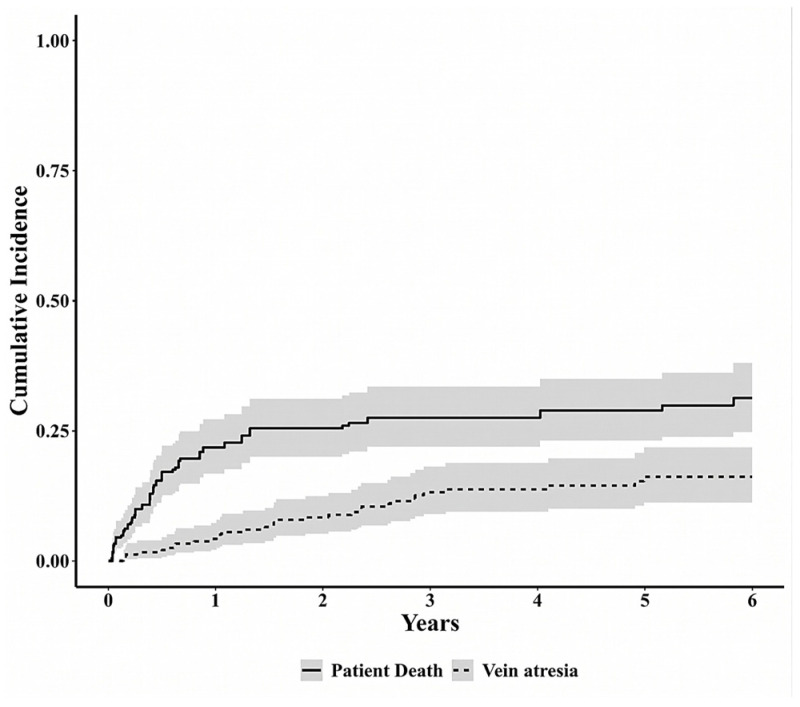
Kaplan–Meier survival analysis. Cumulative incidence modeled to adjust for competing risks of vein attrition due to patient death (solid line) and vein atresia (dashed line). The shaded regions represent 95% confidence intervals (CI).

The cumulative incidence of vein loss increased progressively during the first three years post-diagnosis, followed by plateauing of the survival curve (Figure 2). Of 244 veins, 147 (60%) were lost within three years of diagnosis, including 75 veins (31%) lost secondary to patient death. Vein attrition was greatest within the first year after diagnosis. Of the 147 veins lost, 62 (25%) were lost during the first-year post-diagnosis, 53 of which were due to patient death.

**Figure 3:**
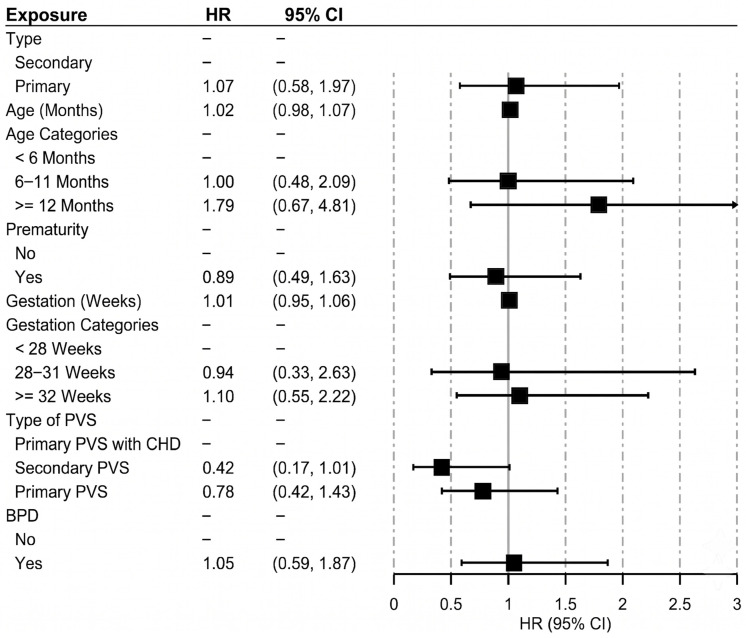
Comparison of patient characteristics between veins lost (N = 147) and veins with recurrent disease that maintained long-term patency for ≥3 years (N = 73). Univariable sub-distribution hazard models, using Fine–Gray regression accounting for multiple veins in each patient. BPD: Bronchopulmonary Dysplasia, CHD: Congenital Heart Disease, APVC: Anomalous Pulmonary Venous Connection (Partial or Total).

Ninety-seven veins (40%) remained patent for at least three years. Of these 97 veins, 73 had recurrent disease, including 55 primary and 18 secondary veins. These 73 veins constituted the cohort of veins with recurrent disease that maintained patency for ≥3 years. Baseline confounding variables which include PVS type, age at diagnosis, prematurity, gestation age, and bronchopulmonary dysplasia were comparable between this cohort and the veins lost during follow-up (Figure 3). Amongst these 73 veins, a median of 2 interventions per year were performed in the first three years after diagnosis, with an IQR of 1–3 in years one and three, and 1–4 in year two. The highest number of reinterventions were performed in the second year, ranging from 1 to 6 interventions per year. After three years, the reintervention rate declined significantly to a median of 1 intervention per year (IOR: 1–3, Figure 4).

## 4. Discussion

This study systematically evaluated the long-term 5-year outcomes of individual pulmonary veins in patients managed within a structured pulmonary vein rehabilitation program. Overall, 244 veins in 107 patients were evaluated, of which 168 (69%) had primary PVS and 142 (58%) involved left-sided veins. Due to limited literature on the long-term outcomes of individual veins in pediatric PVS, survival analysis was first performed and demonstrated progressive vein attrition during the first three years after diagnosis, during which 60% of affected veins were lost. Therefore, three years appeared to represent an inflection point beyond which surviving veins demonstrated sustained patency. Veins with recurrent disease that remained patent at 3 years demonstrated a significant reduction in reintervention rate over the subsequent two years while maintaining continued patency, suggesting stabilization of disease progression. 

Prior studies reporting outcomes of individual veins have been limited by short follow-up durations and failure to account for vein loss attributable to patient death [5,6]. Similar to prior reports, the incidence of vein attrition in our study is high. Importantly, the majority of the veins lost in the first year after diagnosis were due to patient death. Of the 75 veins lost secondary to patient death during the study period, 53 occurred within the first year after diagnosis. Overall, 33 of 107 patients (30.1%) died during the study period, 19 in the first year after diagnosis. Veins that maintained patency beyond three years required fewer reinterventions, indicative of quiescence of disease progression. These findings demonstrate that the first three years following diagnosis represent a crucial therapeutic window during which aggressive protocolized surveillance and intervention strategies are warranted. Maintenance of vein patency throughout this period appears to result in sustained long-term vein patency. Furthermore, sustaining patency of individual veins during this period can translate into improved patient survival by mitigating right ventricular (RV) hypertension which has previously been associated with increased mortality in pediatric patients with PVS [7].

PVS is a chronic and recurrent condition that poses substantial challenges in long-term management. Contemporary treatment strategies employ a multimodal approach incorporating surgical and transcatheter interventions in combination with antiproliferative therapies. Surgical intervention is generally best suited for proximal disease with operative techniques guided by the underlying anatomic substrate [3,8,9]. Because repeated intervention is frequently necessary, transcatheter therapies play a pivotal role in the long-term management and have demonstrated survival benefit [5]. A variety of techniques have been described in prior works, including conventional balloon angioplasty, cutting balloon angioplasty, and stenting with both drug eluting and bare metal stents with varying results [5,10,11]. Furthermore, successful recanalization of atretic veins can also be achieved in as many as 48% of the atretic veins [12]. Molecular signaling pathways such as those involving TGF-β1, and the mTOR pathway, have been postulated to play a role in fibrointimal proliferation characteristic of recurrent PVS [13]. Systemic therapies such as imatinib mesylate and sirolimus targeted at inhibiting these signaling pathways have shown benefit in improving outcomes [6,14].

Utilizing these strategies our institution developed a dedicated pulmonary vein rehabilitation program with standardized surveillance and intervention protocols, as described previously in the manuscript, aimed at minimizing practice variation. Briefly, patients with single-vessel or stable disease are typically evaluated at 3–6 month intervals. Those with multivessel or progressive disease, or with evidence of RV hypertension, undergo more frequent assessments and interventions, typically every 1–3 months. With increasing experience, our approach to systemic sirolimus has evolved from use following stenting of recurrent lesions to earlier initiation after the initial catheterization in patients with severe bilateral multivessel disease. Patients with PVS require a combination of surgery, serial transcatheter interventions, and adjunctive medical therapy throughout their disease course. Our study was therefore designed to evaluate long-term vein outcomes following management within a structured rehabilitation program and to highlight the complementary nature of these treatment modalities rather than isolate the independent effect of individual therapies. We believe these findings support the development of dedicated multidisciplinary pulmonary vein rehabilitation programs that standardize surveillance and treatment strategies to optimize long-term outcomes in pediatric PVS.

The study has several limitations. Its retrospective design can introduce inherent biases and confounding by unmeasurable variables. As a single-center study with small sample size, the independent impact of specific interventions on disease trajectory could not be evaluated. Furthermore, the small number of patent veins precluded subset analysis of reintervention rate stratified by PVS type (primary and secondary). Although our institution has a dedicated pulmonary vein rehabilitation program aimed at standardizing care, variability in procedural decision making during cardiac catheterization may affect outcomes. In addition, adoption of sirolimus use during the study period may have introduced an era effect.

Despite these limitations, this study represents a systematic attempt to characterize contemporary long-term outcomes of individual pulmonary veins in pediatric PVS and to describe modification of disease trajectory within a structured pulmonary vein rehabilitation program. Aggressive surveillance, timely intervention, and mitigation of contributing factors remain imperative to improving both early vein-related and patient outcomes.

## Figures and Tables

**Figure 4:**
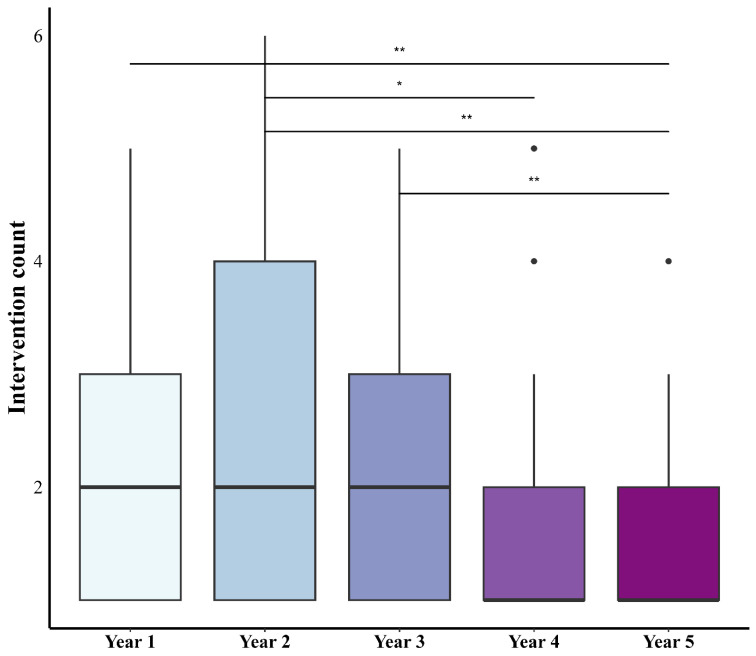
Reintervention rate for veins with recurrent disease that maintained long-term patency for ≥3 years. N = 73, ** *p*-value < 0.01; * *p*-value < 0.05. Mann–Whitney U test for pairwise comparisons between different years. Black dots represent outliers, values falling outside of the ±1.5 × IQR.

**Table 1:** Demographics and patient characteristics.

Characteristic	*N* = 107
Age at Diagnosis (months)	4.1 (2.2–6.4)
Age Categories	
<6 months	77 (72%)
≥12 months	12 (11%)
6–11 months	18 (17%)
Gender	
Female	58 (54%)
Male	49 (46%)
Prematurity	45 (42%)
<28 weeks	15 (14%)
28–31 weeks	15 (14%)
32–36 weeks	15 (14%)
Gestation (weeks)	35 (28–38)
Type of PVS	
Primary PVS with CHD	47 (44%)
Ventricular septal defect	17
Atrial septal defect	6
Complete atrioventricular canal defect	7
Coarctation of aorta ± Ventricular septal defect	5
Tetralogy of Fallot	5
Pulmonary atresia/ intact ventricular septum	2
Hypoplastic left heart syndrome	2
D-transposition of great arteries	1
Tricuspid atresia	1
Ebstein Anomaly	1
Primary PVS without CHD	33 (31%)
Secondary PVS (APVC)	27 (25%)
BPD	48 (45%)
Genetic Syndrome	19 (18%)
Multivessel Disease at Diagnosis	90 (84.1%)
Death	33 (30.1%)
Age at Death (months) ^a^	13.5 (7.6–29.6)
Time to Death (months) ^a^	9.5 (5.6–21.7)

^a^ n = 33. Values are n (%) or median (IQR). Prematurity defined as gestation age < 37 weeks; Time to Death: Time interval between diagnosis and patient death. BPD: Bronchopulmonary Dysplasia, CHD: Congenital Heart Disease, APVC: Anomalous Pulmonary Venous Connection (Partial or Total).

**Table 2:** Characteristics of affected Pulmonary Veins.

Characteristic	*N* = 244
Type	
Primary	168 (69%)
Secondary	76 (31%)
Vein	
Left Upper PV	76 (31.1%)
Left Lower PV	66 (27%)
Right Upper PV	49 (20.2%)
Right Middle PV	25 (10.2%)
Right Lower PV	28 (11.5%)
Veins Lost	147 (60%)
Veins Lost within 1 Year of Diagnosis	62 (25.4%)
Veins Lost to Patient Death	75 (31%)
Veins Lost to Patient Death within 1 Year of Diagnosis	53 (21.7%)
Intervention Count before Vein Loss	3 (2–6)

Values are *n* (%) or median (IQR). PV: Pulmonary Vein.

## Data Availability

The dataset generated and analyzed during the current study are not publicly available due to institutional confidentiality restrictions. However, data is available from the corresponding author upon reasonable request and subject to a formal data sharing agreement.
